# Notes and News

**Published:** 1898-12-10

**Authors:** 


### NOTES AND NEWS.

(Collected and Communicated.)

Vepery, in the Madras Presidency, has just acquired a nice little hospital, the gift of Mr. B. Bauliah Waidu to the Municipality. The advantage of small hospitals is felt in India, as well as in England, where the cottage hospital has now so firm a hold.

The Chester Infirmary is fortunate in one fruitful source of income. The Duke of Westminster devotes the proceeds of fees from visitors who view Eaton Hall to the infirmary. Last year the sum received was no less than £500.

A dispensary is to be opened at Ooregaum in Mysore. Ooregaum is notable for its insanitary condition, and the dispensary may disseminate some useful principles. The hospital assistant who is to be in charge of the dispensary is to attend patients also in the epidemic hospital when established, so he should have enough to do.

A new hospital for Europeans is to be constructed at Calcutta. The whole sum requisite is not immediately forthcoming, so the building is to proceed by instalments and to be used in conjunction with the Presidency General Hospital of Calcutta, which is now undergoing an inquiry into its affairs, great dissatisfaction having been expressed of late.

The Medical, Surgical, and Hygienic Exhibitors' Association held its annual dinner last week. The society, which was founded by the Hon. Treasurer (Mr. F. Rebman) assists both manufacturers and the medical profession by displaying to the one useful novelities, and by giving the others an opportunity of receiving suggestions which they may carry out. Each exhibition has been better attended than the previous one.

Lady Heathcote Amory recently laid the foundation-stone of a new wing at the Tiverton Infirmary. The new buildings are to comprise a large waiting lobby, nurses'-room, operating-room, bathroom, heating apparatus for the entire institution, stores, and sanitary accommodation. In the new wing two wards, containing two beds in each, will be provided for paying patients. Five hundred pounds are still required to meet the expenditure on the undertaking.

An isolation hospital has been erected at Cockrington. The sanatorium is on a hill overlooking the bay of Torquay. The principal wards are on the top floor, each for six patients. A nurses' duty-room is placed between the wards. A third ward is arranged for special patients, accommodation being provided for 14 or 15 patients in all. Coachhouse, washhouse, and mortuary adjoin the main buildings. The cost of the institution has been £1,000, a sum defrayed by Mr. Mallock, who has let the hospital to the District Council for £50 per annum.

Raja Sir Savalay Moodiliar has made a munificent and useful offer to the eight municipal divisions of Madras. This is to establish and maintain in each of these divisions a hospital for such classes of caste Hindus and Mohammedans suffering from the plague as have scruples against entering the Government or municipal hospitals. The President and Plague Commissioner are to select the site, and Sir Savalay will leave the management and supervision in the hands of the municipal executive and the commissioners, himself rendering any assistance and co-operation necessary.

The following candidates having passed the necessary examination have been admitted Fellows of the Royal College of Surgeons in Ireland: Mr. R. Allen, Belfast, M.B., B.Ch., &c., Royal Univ.; Mr. W. J. Corrigan, Cardiff, L.R.C.P.I., L.R.C.S.I., &c.; Mr. A. Cuffe, Essex, L.R.C.S.I., L.A.H., &c.; Miss J. G. Horwood, Aylesbury, L.R.C.P. (Edin.), L.R.C.S. (Edin.), &c.; and Mr. J. O'Callaghan, Pallasgreen, L.R.C.S.I., L.R.C.P. (Edin.), &c. The following candidates passed the primary part of the Fellowship examination: Mr. M. O'Sullivan, Dublin, M.B., B.Ch., &c. (Royal Univ.), and Mr. L. J. Whelan, Dublin, L.R.C.P.I., L.R.C.S.I., &c.

The Melbourne Hospital Saturday and Sunday contributions taken up on October 22nd and 23rd are a little less than last year, £3,685 against £3,707 for 1897. The institution of Hospital Sunday collections began twenty-five years ago, and during that time some £180,000 have been gathered for the charities. In the first year the amount collected was £3,940; ten years later nearly double that amount was collected, and high water was reached in 1888, the year of the "boom," when £12,482 was the sum. Every year after till 1895 the amount decreased, until in that year the sum was £3,900. The next year saw a slight improvement, followed by a further fall last year and this, as bad seasons have been making their effect felt. It is right to remember that during the last few years a number of special appeals have been made. This year the special appeal is for the Children's Hospital, which is building much-needed additions, and has not the wherewithal to meet the contractor's bill at the end of the year, and a number of fêtes and bazaars have been held for different charities—for the Melbourne District Nursing Society and the Austin Hospital for Incurables lately, amongst others—all helping to drain the money from the small population. Meanwhile, every hospital is more or less in debt, and all the charities want money. In the country towns that held collections for their local hospitals on the same days as Melbourne there is a similar tale of decrease in the sums collected.

